# Persuasive antimicrobial stewardship intervention in the context of a KPC outbreak: a controlled interrupted time series analysis

**DOI:** 10.1186/s13756-020-00718-5

**Published:** 2020-04-21

**Authors:** Nuno Rocha-Pereira, Paulo Figueiredo Dias, Sofia Correia, Shirin Shahriari, João Neves, José Teixeira, José Artur Paiva, Carlos Lima Alves, Ana Azevedo

**Affiliations:** 1 Infection and Antimicrobial Resistance Control and Prevention Unit, Hospital Epidemiology Centre, Centro Hospitalar Universitário de São João, EPE, Porto, Portugal; 2 Infectious Diseases Department, Centro Hospitalar Universitário de São João, EPE, Porto, Portugal; 3 Department of Medicine, Faculdade de Medicina da Universidade do Porto, Porto, Portugal; 4 UPCIRA, Centro de Epidemiologia Hospitalar, Centro Hospitalar Universitário de São João EPE, Porto, Portugal; 5 EPIUnit, Instituto de Saúde Pública da Universidade do Porto, Porto, Portugal; 6 Department of Public Health and Forensic Sciences, and Medical Education, Faculdade de Medicina da Universidade do Porto, Porto, Portugal; 7 Angiology and Vascular Surgery Department, Centro Hospitalar Universitário de São João, EPE, Porto, Portugal; 8 Intensive Care Medicine Department, Centro Hospitalar Universitário São João, EPE, Porto, Portugal; 9 Grupo de Infecção e Sépsis, Porto, Portugal; 10 Hospital Epidemiology Centre, Centro Hospitalar Universitário de São João, EPE, Porto, Portugal

**Keywords:** Antimicrobial stewardship, Interrupted time series, Prospective audit and feedback, Antibiotic resistance

## Abstract

**Introduction:**

Antimicrobial resistance is a major public health threat. Antimicrobial stewardship (AMS) is one of the key strategies to overcome resistance, but robust evidence on the effect of specific interventions is lacking. We report an interrupted time series (ITS) analysis of a persuasive AMS intervention implemented during a KPC producing *Klebsiella pneumoniae* outbreak.

**Methods:**

A controlled ITS for carbapenem consumption, total antibiotic consumption and antibiotic-free days, between January 2012 and May 2018 was performed, using segmented regression analysis. The AMS intervention was implemented in the Vascular Surgery ward starting on April 2016 in the context of a KPC outbreak. The General Surgery ward was taken as a control group. Data were aggregated by month for both wards, including 51 pre-intervention and 26 intervention points.

**Results:**

The AMS intervention produced a level change in carbapenem consumption of − 11.14 DDDs/100 patient-days accompanied by a decreasing trend of total antibiotic consumption and stable rate of antibiotic-free days in Vascular Surgery ward. These differences were not apparent in the control group. No differences in mortality or readmission rates between pre-intervention and intervention periods were noticed in any of the groups.

**Conclusion:**

Persuasive AMS interventions on top of previously implemented restrictive interventions can reduce carbapenem consumption without increasing total antibiotic consumption. Starting persuasive AMS interventions in an outbreak setting does not compromise the sustainability of the intervention.

## Introduction

Antimicrobial resistance is a major threat to public health worldwide [1, 2] and there is a global call for action to preserve antibiotics for future generations [3, 4]. Antimicrobials use is deemed inappropriate in at least half of cases and it is well established that antimicrobial use contributes to the development and spread of resistance [5].

In Portugal carbapenem resistance is concerning [6] with several outbreaks of carbapenemase producing *Enterobacteriaceae* registered over the last years and growing proportion of carbapenem resistance noticed in epidemiological surveillance. This kind of bacteria poses major challenges to medical community because of the very few treatment options.

Antimicrobial stewardship (AMS) is one of the key strategies to combat resistance and implementation of such programs is recommended across the globe [7]. These programs aim to improve patient outcomes and safety by improving antimicrobials use and also to reduce emergence of antimicrobial resistance [8–10]. Different types of interventions can be used, namely restrictive and persuasive interventions. The latter includes interventions like audit and feedback, educational outreach and formal or informal local consensus for clinical protocols, all of them aiming at change current behaviours in antibiotic prescription [11, 12]. Both persuasive and restrictive interventions have shown efficacy in reducing antimicrobial consumption [12, 13], duration of therapy, length of stay and also in increasing compliance with guidelines [12, 13]. Some evidence, although less robust, also exists for the role of such strategies in reducing antimicrobial resistance [13]. However, robust evidence supporting different kinds of AMS interventions is still incomplete [14]. To fulfil this gap, in a pragmatic and realistic approach, the use of interrupted time series (ITS) analysis can be useful to evaluate complex interventions used in AMS programs [3, 9, 15].

We conducted a controlled ITS to assess the impact of a set of persuasive AMS strategies implemented in a Vascular Surgery ward in the context of a KPC outbreak.

## Methods

### Setting and design

This study was conducted in Centro Hospitalar Universitário de São João, a 1100-bed tertiary care public teaching hospital in Porto, Portugal. A set of restrictive AMS strategies including formulary restriction, pre-authorization of restricted antimicrobials and automatic stop-orders have been implemented and sustained in all wards for several years. Restricted antimicrobials included carbapenems, quinolones, colistin, daptomycin, linezolid among others. Pre-authorization was performed by the AMS team since 2014 and all restricted antimicrobials were reviewed in a time frame no longer than 24 h in week days. In 2014, following the national legislation, the Infection and Antimicrobial Resistance Control and Prevention Unit (UPCIRA) was created and became responsible for both infection prevention and control and AMS program. Since then, UPCIRA has implemented several persuasive AMS interventions, particularly prospective audit and feedback, in various wards, including Orthopaedics, Plastic Surgery and Burn Unit, Cardiac Surgery and Urology. In April 2016, an outbreak of carbapenemase producing *Klebsiella pneumoniae* was identified in the Vascular Surgery ward. Despite having been identified in April 2016, the first case of the outbreak was traced back to January 2016. The identification of this outbreak prompted UPCIRA to act and alongside with infection control and prevention measures, an analysis of the factors that might have contributed to the occurrence of the outbreak identified excessive use of antibiotics and a high proportion of patients under carbapenem therapy; also, there were no local guidelines for treatment of diabetic foot infections, one of the most common causes of admission to Vascular Surgery ward. Prospective audit and feedback of antimicrobial prescription and production of a local guideline ensued, and both were fully implemented by the end of May 2016. The outbreak ended in July 2016 and in total nine patients in Vascular Surgery ward were affected, with only two of those having infection related with KPC.

We designed a controlled ITS of the period between January 2012 and May 2018 to assess the effect of persuasive AMS strategies on the following endpoints: carbapenems consumption, total antibiotic consumption and proportion of antibiotic-free days.

**Fig. 1:**
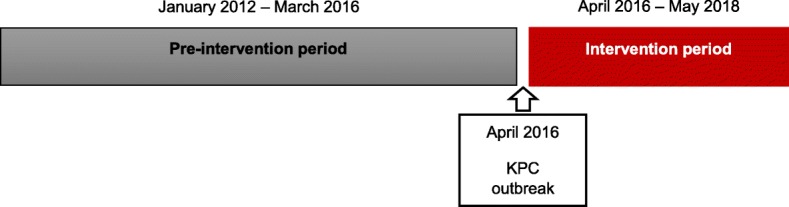
- Schematic representation of the study periods

The period between January 2012 and March 2016 was considered as the pre-intervention period and the period between April 2016 and May 2018 as the intervention period (Fig. 1).

### Intervention

Two different strategies were part of the persuasive AMS intervention: local guideline for diabetic foot infection and prospective audit and feedback on antibiotic prescriptions. The local guideline was produced by a multidisciplinary team including infectious diseases physicians, internal medicine and intensive care physicians, vascular surgeons, endocrinologists, microbiologists, pharmacists and AMS team members. The final version of the guideline was available in May 2016 and was then presented in a lecture to all physicians working in the Vascular Surgery ward. It was also released in the hospital intranet where it became available to all hospital staff. Prospective audit and feedback intervention included the review of all first prescriptions of restricted antimicrobials, all prescriptions of restricted antimicrobials lasting for more than 96 h with the intention of changing to directed therapy and all prescriptions of antimicrobials longer than 8 days. The review was performed weekly by two infectious diseases physicians that were part of the AMS team and was based on the information available in the electronic medical record. The feedback was given in a weekly face-to-face meeting with the prescribing physicians or the physician in charge of each patient. After feedback was given, a clinical discussion between AMS team and prescribing physicians ensued in order to reach a common agreed treatment strategy. The whole-hospital restrictive interventions described above were maintained unchanged during the intervention period.

The control ward, in the General Surgery department, was under the same restrictive AMS interventions described above for the whole hospital but had no defined specific AMS program.

### Data collection

Ward-level data including number of admissions, type of admission (elective vs. urgent), patients’ sex and age, length of stay, 30-day readmission and in-hospital mortality were gathered through an in-house business intelligence platform described elsewhere [16]. Inpatient data on all pharmacy-dispensed antibiotics included in the Anatomical Therapeutical Chemical (ATC) group J01 were expressed as defined daily doses (DDDs)/100 patient-days according to the World Health Organization-ATC/DDD index 2013 [17]. Pharmacy-dispensed antibiotics data in our hospital is corrected considering returned antibiotics. Using the antibiotics’ administration registries, the number of days with no antibiotic was obtained by subtracting the number of days with one or more antibiotic administrations from the number of hospitalization days. The indicator was expressed as a proportion of the entire length of stay. Data were aggregated by month for both wards.

### Statistical analysis

Ward characteristics were described as absolute numbers (number of admissions), proportions or means (length of stay). Monthly averages for the pre- and post-intervention periods were compared for each group using T-test, Wilcoxon rank sum test and χ2 test, as appropriate. Statistical significance was assumed for *p* < 0.05 (two tailed tests).

Carbapenem consumption, total antibiotic consumption and antibiotic free days were analysed separately for Vascular Surgery ward and for the control ward by performing segmented regression analysis of interrupted time series [3].

We tested the change in level of each outcome and the change in slope velocity. Models’ equations are described in the supplementary file S1. We used the STATA command *itsa* which considers segmented linear regression. We ran the models for lag 1 after testing for autocorrelation (no significant autocorrelation was observed at higher orders). Briefly, *itsa* provides the baseline value of the outcome (*β*_0_), the underlying pre-intervention trend (*β*_1_, using time as the predictor) and the change in the outcome level after the intervention (*β*_2_, using a dummy variable defining the intervention) and the change in the slope after the intervention (*β*_3_). The assumptions of normality, homoscedasticity, and linearity were assessed using the Q-Q plot of residuals, plot of residuals against predicted values and plots of residuals against each variable in the regression model.

The data were analysed with STATA version 14.0 and R software version 3.2.2.

## Results

We included in the ITS analysis 51 points (months) before the intervention and 26 points (months) of intervention.

### Ward and patient characteristics

Table 1 summarizes the ward and patient characteristics in both wards in pre-intervention and intervention periods. In the Vascular Surgery ward patients were more commonly males while in control ward there was a predominance of females and this distribution was mantained in pre-intervention and intervention periods.

**Table 1:** General characteristics in Vascular Surgery ward and control group in pre-intervention and intervention periods

	Vascular Surgery			General Surgery		
	Pre-intervention	Intervention	*p*-value	Pre-intervention	Intervention	p-value
**Per month: mean (sd)**						
Admissions (n)	129 (25)	107 (20)	< 0.001	478 (43)	472 (35)	0.575
Elective admissions (%)	74.7 (6.7)	61.4 (11.9)	< 0.001	67.3 (3.7)	64.7 (4.9)	0.013
Men (%)	52.6 (6.2)	57.3 (7.8)	0.005	44.0 (2.6)	42.5 (2.4)	0.013
Age (%)						
< 40	9.5 (3.0)	7.2 (3.5)	0.004	15.4 (1.8)	14.6 (1.5)	0.057
40–64	50.9 (5.8)	45.2 (6.6)	< 0.001	46.4 (2.4)	46.7 (3.0)	0.706
65–74	20.3 (4.0)	25.6 (3.8)	< 0.001	20.1 (1.8)	19.8 (2.3)	0.545
> =75	19.4 (5.3)	22.0 (6.1)	0.058	18.1 (2.0)	18.9 (1.9)	0.078
Average Length of Stay (days)	7.7 (1.5)	9.0 (1.4)	0.001	5.1 (0.5)	5.7 (0.4)	< 0.001
30-days Readmissions (%)	8.0 (2.4)	8.8 (2.6)	0.146	11.5 (1.8)	12.9 (1.7)	0.002
In-hospital death (%)	1.6 (1.3)	2.1 (1.4)	0.167	1.6 (0.6)	2.2 (0.6)	< 0.001

Pre-intervention: January 2012 – March 2016; Intervention: April 2016–May 2018; *sd* standard-deviation

The average number of monthly admissions decreased in the Vascular Surgery ward when comparing pre-intervention and intervention periods while in the control ward no statistically significant change was apparent. The proportion of elective admissions among all admissions decreased in both wards when comparing the pre-intervention and intervention periods, with a reduction of 13.3% in the Vascular Surgery ward.

### Carbapenem consumption

**Fig. 2:**
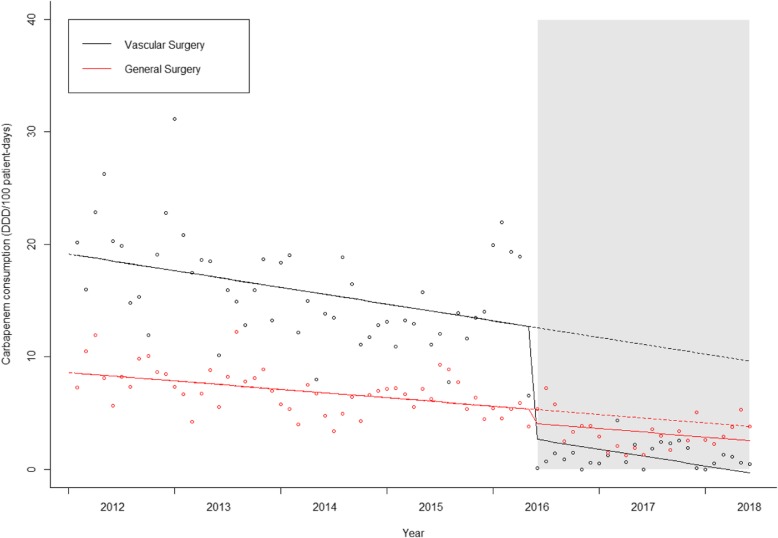
Interrupted time series for carbapenem consumption. Continuous line: predicted trend based on the level change model. Dashed line: counterfactual scenario

In the ITS analysis of carbapenem consumption, both wards had a decreasing trend in pre-intervention period (Table 2 and Fig. 2). In Vascular Surgery ward a level change with intervention implementation was noticed with a decrease of − 11.14 DDDs/100 patient-days (*p* < 0.001) (Table 2). In the control ward no significant level change occurred. In both wards, no significant change in slope during the intervention period, compared to the pre-intervention period, occurred (Table 2).

**Table 2:** Interrupted time-series regression analysis of antibiotic consumption (carbapenems and all antibiotics) and antibiotic free-days in vascular and general surgery wards *(*Adjusted for the proportion of men, age distribution, length of stay and seasonality)

	Vascular Surgery		General Surgery	
	Coefficient (95%CI)	*p*-value	Coefficient (95%CI)	*p*-value
**Carbapenem consumption (DDD/100 PD)**				
Baseline level (*β*_0_)*	*19.38 (16.36; 22.39)*		8..60 (7.47; 9.72)	
Pre-intervention slope (*β*_1_)	−0.16 (− 0.28; − 0.04)	0.008	− 0.05 (− 0.11; 0.00)	0.059
Level change (post intervention) (*β*_2_)	−11.14 (− 16.18; − 6.10)	< 0.001	−1.88 (− 4.18; 0 .43)	0.109
Slope post-intervention (*β*_3_)	0.15 (− 0.01; 0.31)	0.063	0.06 (− 0.07; 0.19)	0.352
**Overall antibiotic consumption (DDD/100 PD)**				
Baseline level (*β*_0_) *	*56.00 (52.22; 59.78)*		*48.04 (48.80; 51.26)*	
Pre-intervention slope (*β*_1_)	0.10 (−0.09; 0.29)	0.302	−0.04 (− 0.18; 0.09)	0.524
Level change (post intervention) (*β*_2_)	2.73 (−7.95; 13.42)	0.611	−0.30 (−4.30; 3.70)	0.880
Slope post-intervention (*β*_3_)	−1.07 (−1.54; −0.61)	< 0.001	0 .45 (0 .21; 0 .68)	< 0.001
**Days without antibiotic (%)**				
Baseline level (*β*_0_) *	*40.87 (38.06; 43.67)*		54.38 (52.33; 56.44)	
Pre-intervention slope (*β*_1_)	0.12 (−0.01; 0.26)	0.076	0.12 (0 .03; 0.20)	0.010
Level change (post intervention) (*β*_2_)	3.18 (−1.86; 8.22)	0.211	−1.46 (−3.56; 0 .62)	0.165
Slope post-intervention (*β*_3_)	0.13 (−0.17; 0.42)	0.395	−0.16 (− 0.31; − 0.02)	0.028

### Total antibiotic consumption

**Fig. 3:**
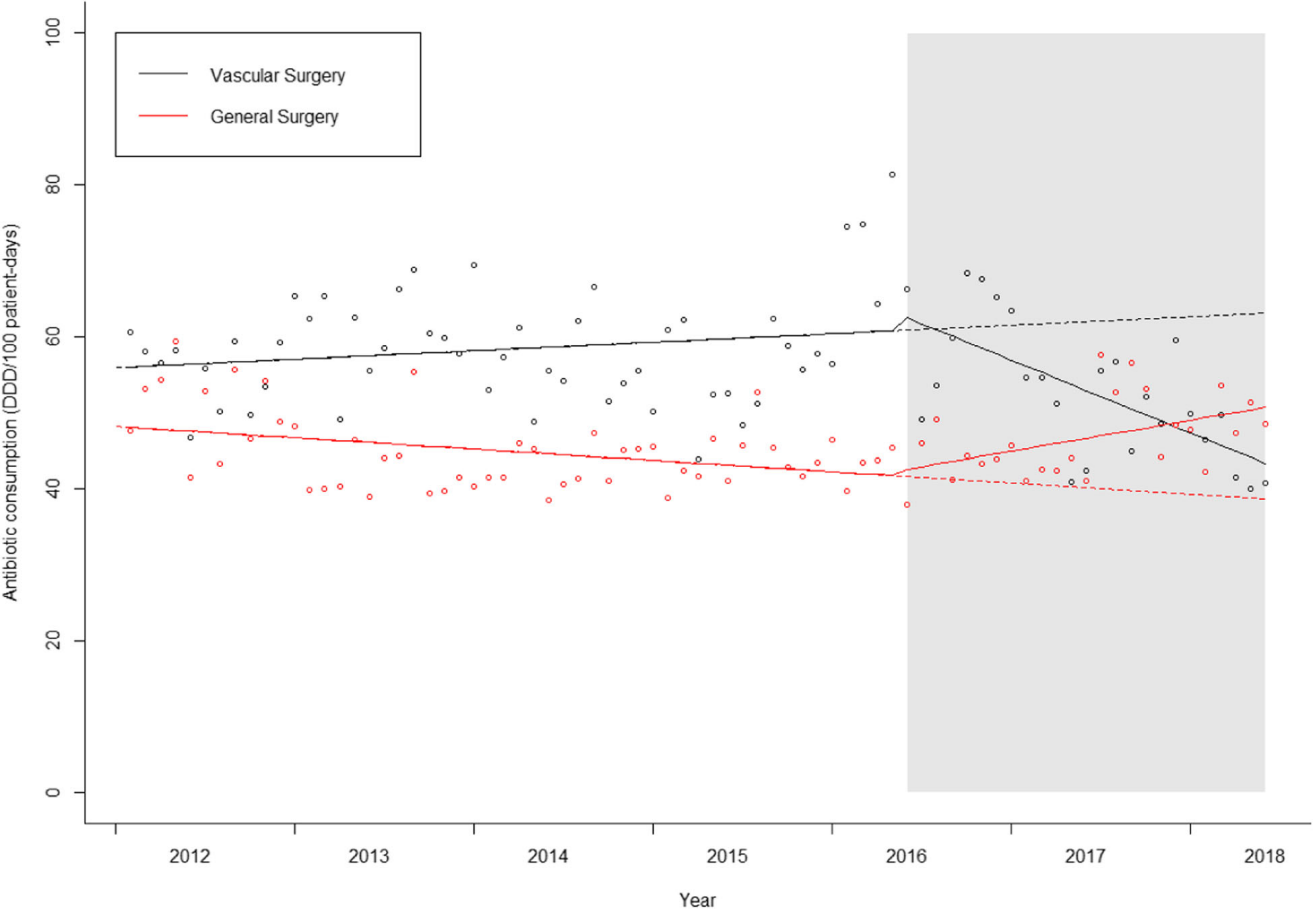
Interrupted time series for total antibiotic consumption. Continuous line: predicted trend based on the level and slope change model. Dashed line: counterfactual scenario

In the ITS analysis of total antibiotic consumption, a significant change in slope between pre-intervention and intervention periods was apparent in both wards (Fig. 3). In the Vascular Surgery ward a significant decreasing trend in intervention period was noticed while in the control ward the opposite occurred (Table 2).

### Antibiotic free-days

**Fig. 4:**
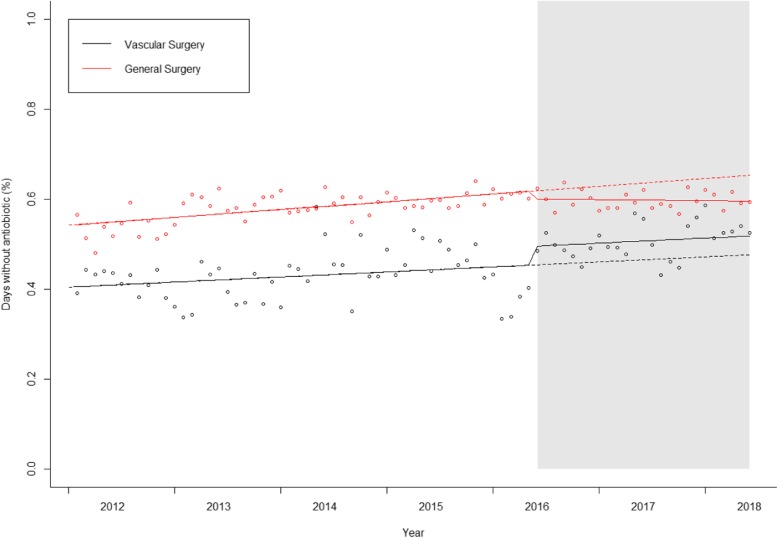
- Interrupted time series for General Surgery department antibiotic-free days. Continuous line: predicted trend based on the level change model. Dashed line: counterfactual scenario

In both wards a positive trend, reflecting an increasing number of antibiotic-free days, was noticed in pre-intervention period (Fig. 4). After intervention implementation a non-significant level change (3.18%, *p* = 0.211) was apparent in the Vascular Surgeryward. During the intervention period, no significant change in slope occurred in Vascular Surgery ward but in the control ward the slope became decreasing (Table 2).

### Other endpoints

Mortality and readmission rates were not significantly different in Vascular Surgery ward in pre-intervention and intervention periods (Table 1). In contrast, in General Surgery ward a stastically significant increase in both endpoints was observed (Table 1). In both wards a significant increase in the average length of stay occurred when comparing pre-intervention and intervention periods (Table 1).

## Discussion

A persuasive AMS intervention, including local guideline production and prospective audit and feedback, was associated with a significant decrease in carbapenem consumption and a reducing trend in overall antibiotic consumption. Both effects were sustained over 2-years duration of the follow-up. Another study performed in our institution, in the Orthpaedics ward, also showed the impact of persuasive AMS interventions (local guideline plus audit and feedback) in antibiotic consumption [18]. Also, several studies in different settings and applying similar persuasive interventions have shown effect in reducing antimicrobial consumption, both globally and for specific antibiotic classes [9, 19]. However, our intervention was designed and implemented in a particularly challenging setting, a KPC outbreak. In this kind of setting usually several interventions are put in place to control the outbreak as rapidly as possible and because AMS effects in terms of resistance are expected on the long run it may be harder to convince healthcare providers of its benefit. Also there are reports that AMS interventions effects usually fade with time with few studies reporting results beyond 24 months [3, 14] and the ones implemented during outbreak settings seem to be specially difficult to sustain once the outbreak is controlled [3]. Our study shows that this kind of intervention may have sustainable results even if the initial design and implementation occur in an outbreak setting. There are concerns that reduction in consumption of some particular classes of antibiotics can result in unintended increase in other classes with similar deleterious ecological effects, the so called “squeezing the balloon effect” [14, 20, 21]. In our study we also measured total consumption of antimicrobials and observed a significant decreasing trend in the intervened ward. This finding suggests that the “squeezing the balloon effect” did not happen in our study. Furthermore, the reduction in carbapenem consumption was accompanied by maintenance in antibiotic-free days in the intervened ward, a finding not replicated in the control ward, where we observed a negative trend in antibiotic-free days.

The main aim of AMS programs is to improve patient care and while reductions in antimicrobial use have been associated with better clinical outcomes, [13, 14] this should not be assumed without direct evidence [3]. Our intervention had no impact in patient mortality or readmission rates, however better indicators such as infection-related mortality would be useful to show that this intervention is safe. Molina et al. [14] showed that a persuasive intervention can even decrease mortality in the long run but the follow-up time of our study is still too short to assess this. There was an increase in length of stay in the intervention period but this increase was noticed in both groups so it can not be attributed to the AMS intervention alone. Also, further patient characteristics needed analysis in order to understand why the length of stay increased. We may speculate that this finding can be related to the significant increase in the percentage of patients admitted non-electively that can have longer and more complicated admissions.

We recognize that our study has some limitations. This is a single centre study which may hamper generalizability; however, it is also true that AMS interventions, as quality improvement initiatives in general, need to be adapted to specific contexts and our study shares evidence on the efficacy of this kind of AMS interventions. We acknowledge that our control group is not perfect because patients’ characteristics and ward dimensions are very different, but it allows to attain the objective of analysing if the differences observed are due to the intervention or due to secular trends in the whole institution [3, 15].. The duration of our study is not long enough to exclude the possible fading of the effect of the AMS intervention but the more than 2 years of intervention are reassuring. Furthermore, our ITS complies with the Effective Practice and Organisation of Care (EPOC) recommendations of recording at least 12 monthly points before and after intervention [3, 22]. Reduction of resistance is one of the major aims of AMS and we did not record data on the evolution of resistance in our study. However, reducing antibiotic consumption can lead to reduced antimicrobial resistance [13] and also, as reported by Geissler et al. [23], longer periods of follow-up are needed to see that kind of effects [2]. As stated above, future analyses of other clinical outcomes like infection-related mortality can be of interest. We may speculate that the fact that our AMS intervention was implemented concurrently with infection control and prevention interventions may have potentiated results, but this seems not be supported by (a) results are sustained over 2 years and (b) excluding a six-month period after the beginning of the intervention did not impact the level change in carbapenem consumption that we report (sensitivity analysis, data not shown).

## Conclusion

Our ITS study shows that persuasive AMS interventions on top of already implemented restrictive interventions can reduce carbapenem and total antibiotic consumption. It also shows that persuasive AMS interventions can be initiated in outbreak settings without compromising the sustainability of the intervention.

## Data Availability

The datasets during and/or analysed during the current study available from the corresponding author on reasonable request.

## Abbreviations

AMS: Antimicrobial stewardship
ATC: Anatomical Therapeutical Chemical
DDDs: Defined Daily Doses
EPOC: Effective Practice and Organisation of Care
ITS: Interrupted time series
KPC: *Klebsiella pneumoniae* carbapenemase
UPCIRA: Infection and Antimicrobial Resistance Control and Prevention Unit
